# Les mycétomes extrapodaux au Sénégal : étude épidémiologique, clinique et étiologique de 82 cas diagnostiqués de 2000 à 2020

**DOI:** 10.48327/mtsi.v2i1.2022.210

**Published:** 2022-02-11

**Authors:** Saër DIADIE, Maodo NDIAYE, Khadim DIOP, Khadim DIONGUE, Joseph DIOUF, Maïmouna SARR, Lamine SARR, Fatimata LY, Mame Thierno DIENG, Suzanne Oumou NIANG

**Affiliations:** 1 Service de dermatologie, Centre hospitalier universitaire Aristide Le Dantec, Dakar, Sénégal; 2 Service de parasitologie, Centre hospitalier universitaire Aristide Le Dantec, Dakar, Sénégal; 3 Service d’orthopédie-traumatologie de l’Hôpital Idrissa Pouye, ex-hôpital général de Grand Yoff (HOGGY), Dakar, Sénégal; 4 Service d’orthopédie-traumatologie, Centre hospitalier universitaire Aristide Le Dantec, Dakar, Sénégal; 5 Service de dermatologie, Institut d’hygiène sociale, Dakar, Sénégal

**Keywords:** Mycétomes extrapodaux, Traitement antifongique, Hôpital, Sénégal, Afrique subsaharienne, Extrapodal mycetomas, Antifungal treatment, Hospital, Senegal, Saharan Africa

## Abstract

**Objectifs:**

Décrire les particularités épidémiologiques, cliniques et étiologiques des mycétomes extrapodaux au Sénégal.

**Méthodologie:**

Étude transversale et rétrospective avec recrutement multicentrique dans quatre services de référence dont deux en dermatologie et deux en orthopédie-traumatologie. Étaient inclus les dossiers de malades présentant un mycétome extrapodal suivis de janvier 2000 à décembre 2020. Les données étaient analysées avec le logiciel SPSS.

**Résultats:**

Nous avons colligé 82 cas représentant 39 % des cas de mycétomes (n = 210). L’âge moyen était de 41,9 ans. Le sexe-ratio était de 3,1. Les professions majoritaires étaient les cultivateurs en activité dans 51 % des cas (n = 33), les éleveurs et les ménagères dans 9 % (n = 6) des cas respectivement. La durée moyenne d’évolution était de 7,5 ans. Les topographies uniquement extrapodales étaient notées dans 84 % des cas (n = 69). Une localisation podale et extrapodale était concomitante dans 16 % des cas (n = 13). Les foyers de mycétomes se répartissaient comme suit: 59 au tronc, 47 aux membres inférieurs, 9 aux membres supérieurs, 1 au cuir chevelu et 1 au cou. L’étiologie était actinomycosique dans 46 % des cas (n = 38), fongique dans 38 % (n = 31). Elle n’a pas été précisée dans 16 % des cas (n = 13). L’atteinte osseuse survenait après 5 ans (p = 0,001) sans lien avec l’étiologie (p = 0,6).

**Conclusion:**

Les mycétomes extrapodaux sont en majorité secondaires à une inoculation directe et leur extension osseuse est exclusivement due au retard diagnostique. Des consultations périodiques en zone d’endémie associées à une formation du personnel de santé résident sont nécessaires pour un dépistage précoce afin d’en améliorer le pronostic.

## Abstract (en)

**Objectives:**

Although the foot remains the main localization of mycetomas, extrapodal attacks, which are rarely studied, are also possible and occur either initially or following an extension. The objectives were to describe the epidemiological, clinical and etiological features of extrapodal mycetomas in Senegal.

**Methodology:**

Cross-sectional and retrospective study with multicentric enrollment in four reference department, two of which are in dermatology and two in orthopedics and traumatology. We included the files of patients with extrapodal mycetoma followed from January 2000 to December 2020. The data were analysed with SPSS software. Any p less than or equal to 0.05 was statistically significant.

**Results:**

We collected 82 cases representing 39% of mycetoma cases (n = 210). The average age was 41.9. The sex ratio was 3.1. Professionally, most patients were active farmers in 51% of cases (n = 33), pastoralists and housewives in 9 % (n = 6) respectively. The mean duration of evolution was 7.5 years. Exclusively extrapodal topographies were noted in 84% of cases (n = 69). Foot and extrapodal locations were concomitant in 16% of cases (n = 13). Mycetoma foci were distributed as follows: 59 in the trunk, 47 in the lower limbs, 9 in the upper limbs, 1 in the scalp and 1 in the neck. The etiology was actinomycotic in 46% of cases (n = 38), fungal in 38% (n = 31). It was not specified in 16% of cases (n = 13). Bone involvement occurs after 5 years (p = 0.001) unrelated to the etiology (p = 0.6).

**Conclusion:**

Extrapodal mycetomas are secondary to direct inoculation. However, extension to bone is exclusively due to diagnostic delay. Periodic consultations in endemic areas combined with training of resident health personnel are necessary for early diagnosis in order to improve the prognosis.

## Introduction

Les mycétomes sont définis comme « tout processus pathologique au cours duquel des agents fongiques ou actinomycosiques d’origine exogène produisent des grains » [5]. Cette maladie tropicale négligée, qui touche avec prédilection le sexe masculin vivant en milieu sylvopastoral, se manifeste par une pseudotumeur cutanée émettant ou pas des grains de couleurs variables [5].

Si la localisation podale est la plus fréquente, d’autres topographies sont possibles soit primitivement, soit par extension à partir du pied [8]. Ces mycétomes extrapodaux (de l’extrémité céphalique à la cheville) sont atypiques et posent un problème diagnostique car ils sont souvent confondus dans notre pratique avec d’autres pathologies tumorales, surtout devant les formes enkystées, non fistulisées. Sur le plan thérapeutique, les difficultés résident dans le choix entre le respect des exigences chirurgicales et la conservation anatomique, fonctionnelle, a fortiori dans les formes fongiques, ou en cas d’atteintes osseuses car il s’agit le plus souvent de zones difficilement résécables [16]. De plus, cette prise en charge se heurte, d’une part à la non-disponibilité de la PCR en cas de grains jaunes ou blancs (obligeant un traitement probabiliste antibactérien) et, d’autre part à l’inaccessibilité des antifongiques dont le posaconazole et l’itraconazole. Eu égard à l’ensemble de ces facteurs, le diagnostic précoce est le seul moyen d’optimiser la prise en charge des mycétomes extrapodaux.

Au Sénégal, deux études ont été réalisées sur les topographies extrapodales remontant à 1995 et 2003 qui, malgré les échantillonnages limités et leur caractère monocentrique, avaient tout de même rapporté la prédominance actinomycosique en accord avec le profil étiologique des mycétomes et la fréquente atteinte de la jambe [7, 18].

Alors qu’une transition étiologique est décrite par la plupart des séries sénégalaises de mycétomes réalisées dans la décennie montrant une prédominance actuelle des formes fongiques, nous avons jugé opportun de réaliser une mise au point sur les mycétomes extrapodaux [13, 19].

L’objectif principal était de décrire les aspects épidémiologiques, cliniques et étiologiques des mycétomes extrapodaux au Sénégal. De plus, nous avons cherché à déterminer le délai de survenue des atteintes osseuses et à savoir si l’étiologie joue un rôle déterminant dans l’envahissement osseux.

## Patients et Méthodes

Nous avons procédé à une étude rétrospective avec recrutement multicentrique mené dans quatre services de référence dont deux en dermatologie et deux en orthopédie traumatologie: services dermatologie-vénéréologie du CHU Aristide Le Dantec (HALD) et de l’Institut d’hygiène sociale de Dakar, services d’orthopédie-traumatologie de l’HALD et de l’hôpital général de Grand Yoff. Étaient inclus tous les dossiers de patients présentant un mycétome et suivis dans ces sites de janvier 2000 à décembre 2020 (21 ans). Nous avons étudié les caractéristiques des localisations extrapodales. Le diagnostic portait sur les éléments:

- épidémiologiques, notamment la provenance ou le séjour dans une zone d’endémie;
- cliniques, en particulier une tuméfaction polyfistulisée ou enkystée avec allégation ou mise en évidence de grains par le praticien;
- parasitologiques et/ou histologiques.

L’étiologie fongique ou actinomycosique était établie selon la couleur des grains, l’examen parasitologique, la PCR et les arguments thérapeutiques dans certains cas.

Les données étaient recueillies par un questionnaire et analysées par le logiciel SPSS. Le seuil de signification était 0,05.

## Résultats

Nous avons recensé 82 patients présentant des mycétomes extrapodaux, soit 39 % des cas de mycétomes observés (n = 210) pendant la période d’étude. L’âge moyen des patients était de 41,9 [14-85] ans avec une médiane de 37,5 ans. Le sexe-ratio était de 3,1. Les patients venaient majoritairement des régions de Diourbel (165 km à l’est de Dakar) avec 18 % (n = 14) et Louga (225 km au nord de Dakar) représentant 17 % (n = 23), puis de Thiès (75 km à l’est de Dakar) et Saint-Louis (294 km au nord de Dakar) soit 12 % (n = 9) chacune.

La profession a été renseignée dans 81 % des cas (n = 67). Les activités majoritaires étaient les cultivateurs en activité pour 51 % (n = 33), les éleveurs et les ménagères dans 9 % (n = 6) des cas respectivement.

La durée moyenne d’évolution était de 7,45 ans [6-40 ans] avec une médiane de 6 ans. La moitié des malades avaient consulté avant 5 ans.

**Figure 1:**
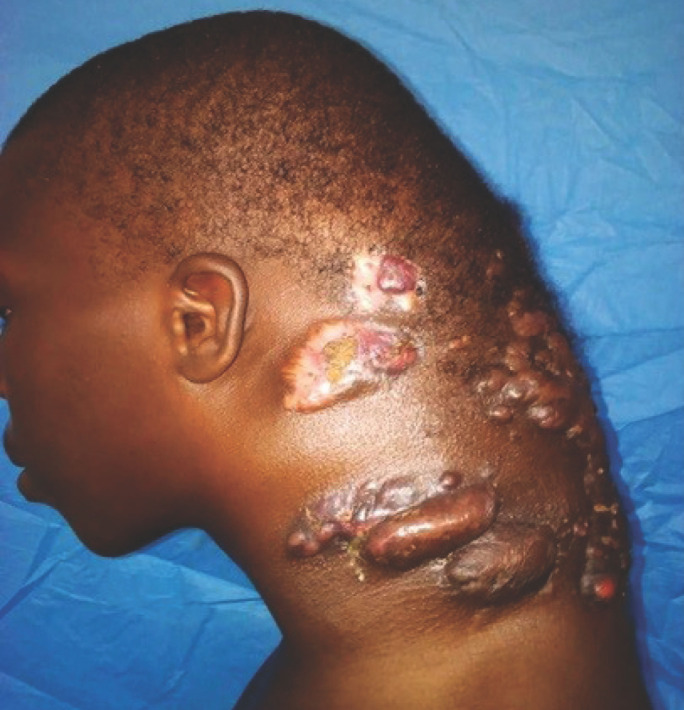
Mycétome cervical actinomycosique déformant en cou de dragon Actinomycotic cervical mycetoma deforming into dragon neck

Les topographies uniquement extrapodales étaient notées dans 84 % des cas (n = 69). L’un d’entre eux siégeait au cou (Fig. 1). Une localisation podale et extrapodale étaient concomitantes dans 16 % (n = 13) dont 1 cas d’inoculation bifocale (dos de la main et pied). Les 12 autres cas étaient consécutifs à une extension à partir d’un foyer primitivement podal.

La répartition des 117 foyers de mycétomes chez nos 82 patients est détaillée dans le Tableau I.

**Tableau I:** Répartition des localisations des mycétomes extrapodaux Distribution of extrapodal mycetoma locations

Partie du corps	Nombre de localisations	Siège	
Membre inférieur	47	Genou Cheville Jambe Cuisse	n = 12 n = 8 n = 13 n = 14
Tronc	59	Thorax Fesse Pubis Périnée Lombe Dorso-lombaire Aine	n = 7 n = 16 n = 12 n = 12 n = 7 n = 2 n = 3
Membre supérieur	9	Main Épaule Poignet Aisselle Bras	n = 5 n = 1 n = 1 n = 1 n = 1
Cuir chevelu	1		n = 1
Cou	1		n = 1

**Figure 2:**
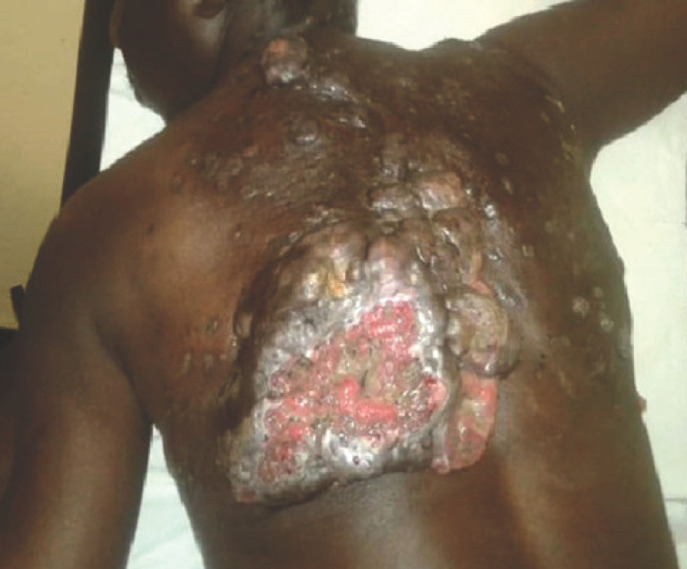
Mycétome dorsal, tumoral, fongique, à grains noirs Dorsal, tumoral and fungal mycetoma with black grain

La présentation clinique a été renseignée dans 73 cas. Le mycétome était inflammatoire, fistulisé dans 55 % des cas (n = 40), tumoral dans 44 % (n = 32) (Fig. 2) et encapsulé dans 1 % (n = 1).

**Figure 3:**
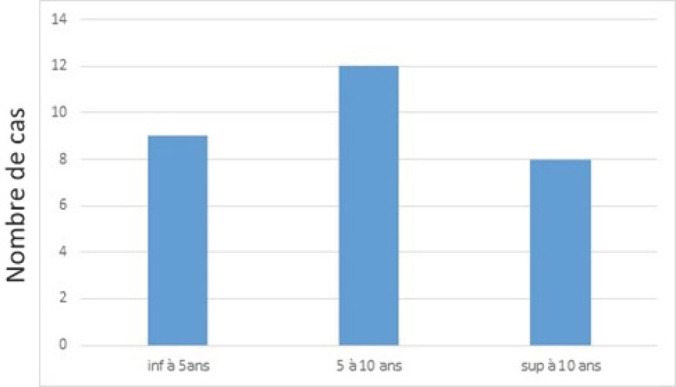
Répartition des atteintes osseuses selon la durée d’évolutionz Distribution of bone damage according to the duration of evolution

Une atteinte osseuse a été notée dans 35 % des cas (n = 29) dans des délais précisés à la Figure 3.

L’envahissement osseux était consécutif à une atteinte de la jambe dans 7 cas, du genou dans 6 cas, de la fesse et de la cheville dans 4 cas chacune. Trois cas d’atteinte périnéale et 2 cas de mycétomes de la main ont présenté des complications osseuses.

L’étiologie était actinomycosique dans 46 % des cas (n = 38) et fongique dans 38 % (n = 31). L’étiologie n’a pas été précisée dans 16 % (n = 13) des cas.

**Figure 4:**
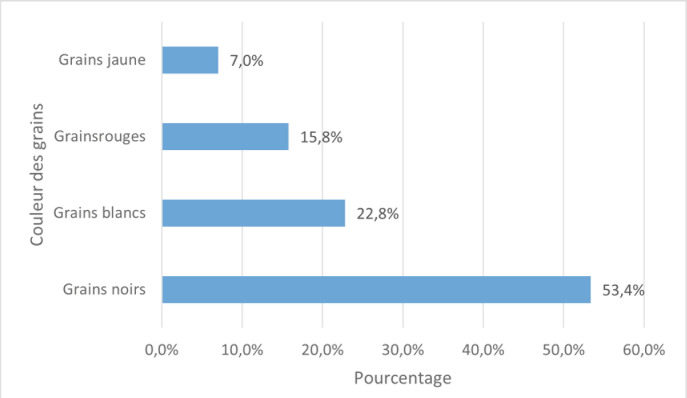
Répartition des mycétomes extrapodaux selon la couleur des grains identifiés Distribution of extrapodal mycetoma according to the identified grain color

La couleur des grains était renseignée chez 57 malades (Fig. 4). Les grains blancs étaient bactériens dans 12 cas. L’étiologie du treizième cas n’a pas été précisée. Les grains jaunes étaient tous bactériens. Dans les cas où l’identification n’a pas été faite devant des grains jaunes ou blancs, l’origine bactérienne a été retenue devant la guérison sous antibiotiques.

**Figure 5:**
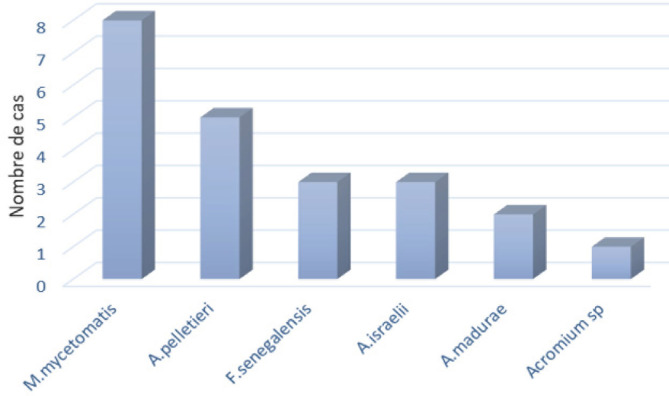
Répartition des mycétomes extrapodaux selon l’agent pathogène identifié Distribution of extrapodal mycetomas according to the identified pathogen

Parmi les cas où l’espèce a été identifiée (n = 22), il existait autant d’espèces fongiques qu’actinomycosiques (Fig. 5).

Parmi les cas de mycétomes extrapodaux dont l’étiologie a été identifiée, nous avons observé plus de formes actinomycosiques (n = 38) que fongiques (n = 31). Par contre, s’agissant des localisations podales, les causes fongiques étaient prédominantes (fongique, n = 61; bactérien, n = 55) (Tableau II). Les atteintes osseuses étaient consécutives à une topographie podale dans 66 % des cas, alors que les localisations extrapodales n’en constituaient que 22 %. Il existait une corrélation entre la topographie et l’atteinte osseuse (p = 0,006).

**Tableau II:** Répartition des localisations des mycétomes selon l’origine fongique ou actinomycosique Distribution of mycetomas location according to fungal or actinomycotic origin

	Mycétomes podaux	Mycétomes extrapodaux
Fongiques	61	31
Actinomycosiques	55	38

Nous avons observé une fréquence plus élevée des atteintes osseuses après 5 ans (p = 0,001). Cependant, l’étiologie n’avait pas d’influence sur la survenue de l’atteinte osseuse quelle que soit la durée d’évolution (p = 0,6).

## Discussion

Notre échantillon nous semble représentatif de la distribution des mycétomes au Sénégal en fonction du climat, sinon de l’incidence. En effet, le recrutement est non seulement multicentrique, mais est également effectué dans les services de référence de prise en charge des mycétomes au Sénégal. Ce qui nous permet d’établir des extrapolations à l’échelle nationale.

Nous avons rapporté 82 cas de mycétomes extrapodaux représentant 39 % des cas de mycétomes avec une fréquence de 4,2 cas annuels. Ces mycétomes touchent avec prédilection l’homme d’âge mûr, de 40 ans en moyenne. Si ces résultats confirment la prédominance des localisations podales, ils corroborent les constats déjà établis par des études réalisées au Sénégal durant les trente dernières années. D’abord en 1992, Ndoye et al estimaient leur prévalence à 35 % [14]. Trois ans plus tard, une série rapportée par Ndiaye montrait que les mycétomes extrapodaux constituaient 46,8 % des cas au Sénégal [11]. Ceci était similaire aux résultats de Soumaré en 2003 qui, dans une étude sur 20 ans colligeant 156 cas de mycétomes notait une prévalence de 42 % [18]. À la même période, Dieng et al rapportaient un taux de 38,5 % sur 130 cas de mycétomes [8].

L’amélioration et la guérison clinique sous traitement antibiotique ont été le principal argument rétrospectif ayant permis de retenir la nature actinomycosique dans les cas de mycétomes à grains blancs ou jaunes (qui peuvent être bactériens ou non). Dans notre pratique, devant le long délai de rendu de résultat, un traitement antibactérien est débuté dès lors que les grains sont de couleur blanche ou jaune, d’autant plus que les explorations mycologiques classiques (examen direct et culture) ne sont pas toujours contributives. Ce n’est qu’en 2021 que des sondes PCR ont été disponibles au laboratoire de parasitologie du CHU Aristide Le Dantec.

Les mycétomes extrapodaux étaient majoritairement d’origine actinomycosiques (46,4 %). Nous confirmons les conclusions de Dieng et al qui avaient décrit les formes bactériennes dans 60 % des cas des mycétomes extrapodaux [7]. Cependant, nos résultats ne sont pas en phase avec le profil étiologique récent des mycétomes au Sénégal. Les récentes observations établissent une prédominance d’agents fongiques. À titre illustratif, Ndiaye en 2011, Sow en 2020 et Badiane la même année avaient décrit les eumycétomes dans 70 %, 47.2 % et 61,1 % des cas respectivement [1, 13, 19]. Il existe ainsi une évolution étiologique notable, car les premiers travaux réalisés notaient une prédominance d’agents actinomycosiques (68,4 %) [7, 11, 12]. Ces derniers sont retrouvés avec prédilection dans des zones de pluviométrie située entre 500 et 800 mm, contrairement aux agents fongiques qui sévissent dans des zones moins pluvieuses (250-500 mm) [15]. Le changement climatique, occasionnant une modification des isohyètes avec une baisse de la pluviométrie, est l’une des raisons qui expliquerait la modification du faciès des mycétomes avec une tendance majoritaire des eumycétomes dans les zones de très faible pluviométrie.

Les actinomycètes ont été pendant longtemps considérés plus ostéophiles que les eumycètes. Toutefois notre étude suggère que l’agent pathogène n’influe pas dans la survenue de l’extension osseuse (p = 0,08). En effet, cet envahissement osseux était exclusivement lié à la durée d’évolution (au-delà de 5 ans) quel que soit l’agent pathogène. *Madurella mycetomatis,* agent fongique, était davantage retrouvé dans les cas d’extension osseuse.

La prévalence des mycétomes extrapodaux est plus élevée au Sénégal comparée aux pays d’Afrique où les études montrent une prédominance des localisations podales. Estimées à plus de 35 % au Sénégal, les localisations extrapodales représentent 17.3 % des cas au Niger [4], 18,5 % au Mali [17] et 31,2 % en Somalie [3]. Il en est de même au Soudan, pays de référence tant par la fréquence et la prise en charge que par la recherche scientifique, où les localisations extrapodales ne représentaient que 24 % sur une série de 6 792 cas [10]. Certaines habitudes (port de fagots, couchage à même le sol lors des pauses et satisfaction des besoins naturels dans les herbes) pourraient expliquer ces différences. En effet, ces comportements exposent certaines régions anatomiques d’habitude inaccessibles aux traumatismes inoculateurs, à l’occasion de piqûres d’épines. La prédominance des atteintes du tronc et la fréquence des atteintes fessières chez nos malades constituent une illustration de ces pratiques. De même, le port de fagots au niveau du thorax constitue le principal mécanisme évoqué par les auteurs mexicains expliquant la prédominance des localisations thoraciques au Mexique [2].

Les mycétomes extrapodaux touchent électivement le sujet d’âge mûr de sexe masculin d’environ 42 ans. Ces résultats n’offrent aucune particularité et s’apparentent aux études déjà menées montrant un âge de survenue autour de 40 ans [3,4,8,17]. La prédominance du sexe masculin s’expliquerait, d’une part par l’activité agricole ou pastorale (60 %), et d’autre part par des facteurs génétiques qui exposeraient davantage l’homme. Quoique soumises aux mêmes conditions environnementales à travers les activités rurales, les femmes demeurent tout de même moins infectées. Ceci suggère l’implication de facteurs hormonaux dans la survenue des mycétomes. L’aggravation de la maladie chez des femmes pendant la grossesse et son caractère exceptionnel avant la puberté plaident en faveur du rôle possible des hormones dans la genèse des mycétomes [2]. Nous pensons qu’il existe un facteur prédisposant au développement de la maladie, car toutes les personnes exposées aux mêmes conditions environnementales et climatiques ne développent pas obligatoirement la maladie. Ceci est d’autant plus vraisemblable que récemment, la mutation du gène de la chitotriosidase, enzyme macrophagique qui joue un rôle dans la phagocytose et la digestion, a été mise en évidence au cours des mycétomes à *M. mycetomatis.* Les individus qui présentent des mutations génétiques aboutissant au déficit de l’activité du chitotriosidase sont plus exposés aux eumycétomes [20].

Nous confirmons que le retard diagnostique au-delà de 5 ans est un facteur prédictif d’atteinte osseuse (p = 0,001) conformément à l’étude de Ndiaye [6]. Cependant, l’étiologie fongique ou actinomycosique ne semble pas influer sur la survenue de cette complication (p = 0,08). Ce retard diagnostique est classiquement décrit au Sénégal où le délai est estimé entre 6 et 9 ans [7, 13, 18] ainsi que dans d’autres pays africains [3,4,10,17]. Cette longue période d’errance diagnostique est consécutive à plusieurs facteurs: l’évolution insidieuse de la maladie souvent indolore et le manque de moyens financiers pour la plupart de nos malades qui travaillent dans le secteur informel. De plus, la maladie est méconnue par le personnel soignant. Les malades ont ainsi recours aux marabouts et tradipraticiens parce qu’ils adhèrent fortement au diagnostic d’ensorcellement et procèdent à des sacrifices nécessitant des moyens financiers considérables. Ceci aboutit à une altération de la qualité des soins ultérieurs pour plusieurs raisons: d’une part, la progression destructrice de la maladie et, d’autre part l’épuisement financier consécutif au long itinéraire thérapeutique et au coût élevé des antifongiques [6, 9].

Ainsi, seul un diagnostic et une prise en charge précoces permettront d’améliorer le pronostic de la maladie. Dans un premier temps, il est urgent que les dermatologues effectuent des consultations avancées dans les zones dites endémiques afin de dépister précocement les cas de mycétomes. Ensuite, des formations médicales continues doivent être effectuées à l’endroit du personnel soignant qui exerce dans des zones rurales afin de les rendre capables de reconnaître la maladie dans toutes ses formes. Ces actions nécessitent une volonté politique, l’intégration et la consultation des spécialistes dans les prises de décision en particulier dans les maladies tropicales négligées (MTN). En plus d’être urgentes, elles doivent s’inscrire dans le temps afin de limiter le fardeau, car les sujets sont non seulement jeunes, mais sources de revenus financiers pour des familles généralement pauvres. Cette stratégie est d’autant plus utile qu’on note une fréquence de plus en plus accrue des formes fongiques dont le traitement efficace n’est accessible que par la chirurgie d’exérèse au stade de début [13, 19].

## Conclusion

Les mycétomes extrapodaux sont fréquents et tardivement diagnostiqués au Sénégal. Ce retard est le principal facteur de survenue des lésions osseuses. Des mesures d’urgence doivent être prises. Il s’agira d’effectuer des consultations avancées dans les zones dites endémiques afin de dépister précocement les cas, puis de promouvoir des formations médicales continues à l’endroit du personnel soignant résident. De même, le diagnostic par PCR doit être davantage disponible afin de faciliter l’identification des grains, surtout en cas de couleur jaune ou blanche, car l’histopathologie et l’étude parasitologique ne sont pas toujours contributives.

## Liens d’intérêts

Les auteurs ne déclarent aucun lien d’intérêt.

## Contribution des auteurs

Saer DIADIE, Maodo NDIAYE et Maïmouna SARR ont participé dans la conception, l’exécution et la rédaction de l’étude.

Lamine SARR, Fatimata LY, Mame Thierno DIENG, Khadim DIOP, Khadim DIONGUE et Joseph DIOUF ont participé au diagnostic des cas.

Suzanne Oumou NIANG a participé à la relecture et validation du manuscrit
